# Changes in quality of life throughout the illness trajectory of older adults with cancer: a systematic review

**DOI:** 10.1093/oncolo/oyaf223

**Published:** 2025-07-23

**Authors:** Chelsea Vinckier, Kim de Nooijer, Tinne Smets, Helena Du Cheyne, Lore Decoster, Lieve Van den Block

**Affiliations:** End-of-Life Care Research Group, Vrije Universiteit Brussel (VUB), Brussels, Belgium & Ghent University, Ghent 9000, Belgium; End-of-Life Care Research Group, Vrije Universiteit Brussel (VUB), Brussels, Belgium & Ghent University, Ghent 9000, Belgium; End-of-Life Care Research Group, Vrije Universiteit Brussel (VUB), Brussels, Belgium & Ghent University, Ghent 9000, Belgium; End-of-Life Care Research Group, Vrije Universiteit Brussel (VUB), Brussels, Belgium & Ghent University, Ghent 9000, Belgium; Department of Medical Oncology, University Hospital Brussel (UZ Brussel), Translational Oncology Research Center (TORC) team laboratory for medical and molecular oncology, Vrije Universiteit Brussel (VUB), Brussels 1090, Belgium; End-of-Life Care Research Group, Vrije Universiteit Brussel (VUB), Brussels, Belgium & Ghent University, Ghent 9000, Belgium

**Keywords:** quality of life, aged, neoplasms, longitudinal studies, trends, systematic review

## Abstract

**Background:**

Older adults with cancer often experience frailty and comorbidities, potentially impacting their quality of life. Knowledge on quality-of-life changes throughout the cancer trajectory is important to set feasible expectations in interventions or trials.

**Aim:**

This systematic review synthesizes existing knowledge on quality-of-life changes in older adults with cancer throughout their illness trajectory and explores potential individual, relational, community, and societal factors associated with these changes.

**Method:**

We systematically searched PubMed, Embase, and PsycINFO. We synthesized characteristics and outcomes of all studies reporting on quality-of-life or well-being changes in people aged 65+ with cancer. We used Bronfenbrenner’s Social Ecological Model to categorize associated factors. We followed PRISMA guidelines and registered in PROSPERO (CRD42024566815).

**Results:**

We included 22 studies. Studies varied in characteristics of the cohorts and timing of the measurements and were often lacking clear quality-of-life conceptualizations. All studies used a quantitative design, except 1 mixed-methods study. Eight of 9 studies with follow-up moments of 12 months or more reported stable quality-of-life scores. All 3 studies examining the last year of life found quality-of-life declines. Five other studies reported a decline during treatment; in 4 studies followed by an increase. Comorbidities, older age, and mobility problems were most frequently associated with declining quality of life. Relational, community, and societal level factors were rarely studied.

**Conclusion:**

Despite the methodological heterogeneity between studies, we identified trends in quality-of-life changes across the illness trajectories of older adults with cancer, particularly declining trends during treatment and end-of-life periods and stable trends in long-term follow-up.

Implications for practice Clinicians caring for older adults with cancer should be aware that, while some experience changes in their quality of life, this is not the case for all older adults, as we found several longitudinal studies showing stable trajectories. Periods that merit attention include treatment and end-of-life periods, both found to be related with declines in quality of life or well-being. Support during these periods seems particularly crucial.

## Introduction

### Background

Cancer mostly affects older adults, with an estimated 60% of the new diagnoses in 2020 occurring in people aged 65 or older.^1^ Next to the direct impact of cancer, older adults with cancer often suffer from frailty, comorbidities, and declining health.^2^ Because of this complexity of comorbidities and frailty, cancer and its treatment can have a different impact on older adults’ quality of life compared to younger people and can lead to worsening health, functional problems, and social isolation.^2^^,^^3^ Therefore, in older adults, supporting and enhancing quality of life is often considered to be just as important as life-prolonging treatment and care.^4^^,^^5^ Quality of life—and more specifically health-related quality of life—has also increasingly been used as an outcome in cancer trials and other health research.^4^^,^^5^ Additionally, the importance of well-being and therefore its utilization in public health has been growing, although it faces difficulties with operationalization.^6^

Although the quality of life and well-being of older adults with cancer are considered crucially important, no research has synthesized how quality of life or well-being changes over time in this population. Lacking this knowledge, researchers do not know what can be expected when quality of life is targeted in interventions. Hence, current clinical trials or interventions targeting older adults with cancer might be setting inappropriate outcomes or have unrealistic expectations about possible effects. Studies within the general population have shown that, throughout life, well-being follows a U-shaped trajectory, with the lowest well-being scores observed in people between 45-54 years old and relatively high scores among people aged 70 or older.^7^ Studies on adults with cancer across all age groups have shown conflicting findings on quality of life. While some research reports stable quality-of-life scores at specific points in the disease trajectory (eg, during chemotherapy), others indicate either improvements or declines.^8^^,^^9^ Lee et al. (2022) found that adults with advanced cancer with low educational levels are more likely to have a progressively declining health-related quality of life than patients who are higher educated.^10^ Higher age and being male were also strong predictors of a declining health-related quality of life in the last year of life.^10^ Other studies have found that comorbidities^11^ in adults with advanced cancer, economic burden,^8^ and depressive symptoms^8^ in newly diagnosed adults with breast cancer are predictors of low quality of life.

As cancer affects older adults differently than younger people,^3^ it is impossible to generalize conclusions from adult cancer patients or general populations to older adults with cancer. A 2009 review of Ballinger et al. suggested that older adults with breast cancer are perhaps better equipped psychologically to cope with treatments than their younger counterparts.^12^ However, this review mostly compared quality of life during and after different breast cancer treatments, excluding quality-of-life trajectories of patients with other cancer types and patients not receiving treatment. Since then, several studies in different populations of older adults with cancer have been performed, with seemingly contradictory results. For example, Esbensen et al. aimed to investigate changes in quality of life among older adults with cancer (all types) at 3 and 6 months after diagnosis and found that quality of life remained stable over 6 months, whereas the study of Kirkhus et al. (2019) reported declining quality-of-life scores at 2 and 4 months after diagnosis among older multiple myeloma patients.^13^

The aim of this systematic review is to synthesize existing knowledge of changes in quality of life of older adults with cancer throughout their illness trajectory and to explore potential individual, relational, community, and society level factors associated with these changes.

## Methods

This systematic review followed the Preferred Reporting Items for Systematic reviews and Meta-Analyses (PRISMA) statement.^14^ The protocol was registered in the international Prospective Register of Systematic Reviews (PROSPERO) database (ID: CRD42024566815).

### Eligibility criteria

Publications eligible for inclusion reported on

- People aged 65 years or older, AND
- People with an active cancer (any stage, any diagnosis, not in remission), AND
- Quality of life and/or well-being, AND
- Studies with a quantitative and/or qualitative research design, AND
- Longitudinal, prospective or trajectory studies, and/or containing a minimum of 3 measurement moments/observations AND
- Changes in quality of life and/or well-being throughout the illness trajectory, AND
- Articles written in English.

The following publications were excluded:

- Reviews or meta-analysis, conference abstracts, editorials, book chapters, dissertation theses, study protocols, pre-post-test designs, case-reports, feasibility studies, OR
- Articles exclusively focused on testing the validity of a quality-of-life or well-being instrument, OR
- Articles reporting on 1 specific aspect of quality of life or well-being, such as fatigue, symptoms, sleep, OR
- Articles exclusively focused on cancer treatments or diagnostic, therapeutic, or other health-related interventions, using quality of life and/or well-being as an outcome.

### Information sources and search strategy

The PubMed, Embase, and PsycINFO databases were searched for relevant articles. The search was conducted on December 2, 2024. Search terms were developed by CV and KDN in collaboration with a librarian of the Vrije Universiteit Brussel (VUB) and the author group. Search terms were focused on older people (aged 65 or older), cancer, quality of life/well-being, and illness trajectories/longitudinal studies (and synonyms) (Table S1). We also searched the reference lists of relevant articles and systematic reviews.

### Selection process

**Figure 1.**
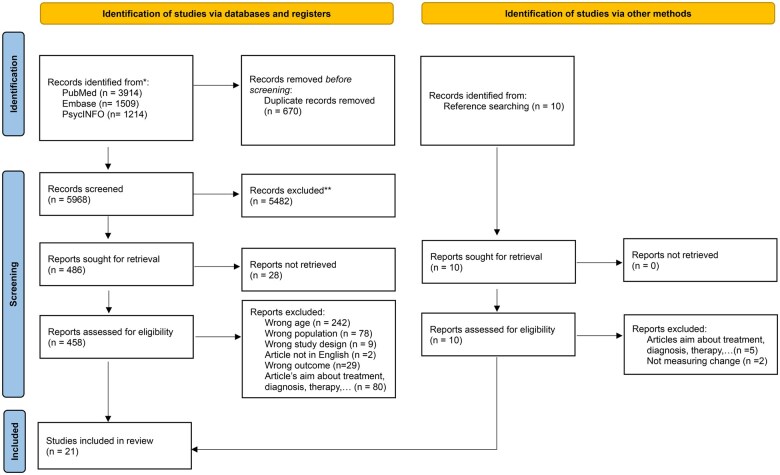
PRISMA flowchart of study selection process.

CV screened all articles by title and abstract, and a second reviewer HDC independently screened 21% of the articles (1,269 articles). We used Rayyan, a web and mobile app guiding the screening of articles for systematic reviews.^15^ In total, there were 103 inconsistencies between CV and HDC. These were discussed and resolved in discussion with a third reviewer (TS). After title and abstract screening, 458 full texts were screened by CV and 20% by HDC (Figure 1).

### Data extraction and synthesis

CV extracted relevant data from all included articles and HDC from 10%. Inconsistencies were discussed among the 2 researchers and conflicts were discussed with a third reviewer (T.S.). We developed extraction tables in MS Word. We extracted the following data: author, year, country, main study objectives, method, design, sample size, age (minimum and mean), gender, cancer type, cancer phase, quality of life or well-being conceptualization, instrument(s) used to measure quality of life or well-being, number of measurements, statistical analysis (within subject analyses), retention rate, quality-of-life score per measurement moment, reported change in quality of life, and the factors associated with quality-of-life changes.

A meta-analysis was not possible due to the heterogeneity of the included studies (variation in quality-of-life instruments used, timepoints, population, etc.). We present an overview of the included studies, detailing their characteristics, study population, and quality-of-life conceptualizations. A pie-chart illustrates the quality-of life operationalizations and changes in quality of life are summarized in a table. In this table, we plotted the times where quality of life was measured, and quality-of-life trends are visually represented using upward and downward arrows or equal signs. Studies were grouped based on their baseline measurement point, and quality-of-life trends were interpreted by quantifying the number of upward or downward arrows or equal signs.

We synthesized the factors associated with changes in quality of life using the Social Ecological Model (SEM) by Urie Bronfenbrenner^16–19^. A first level concerns the individual level, including demographic characteristics, beliefs, norms, and behaviors of the person. A second level is relational. This contains family, friends, partners, and other close relations. The third level—organizational/community—includes the resources, social norms, neighborhoods, environmental characteristics, health services, and organizations near the person. The fourth level—societal—refers to policies and laws impacting people’s health and well-being.^19^

### Quality assessment

CV evaluated the quality of all the included articles as well as the risk of bias in the studies or bias in reporting, using the QualSyst tool,^20^ and HDC evaluated 10%. This tool includes a checklist to assess the quality of both qualitative and quantitative studies with different designs. QualSyst scores range from 0 to 1, with 1 being the highest possible score (ie, the least bias and best quality). We used a relatively conservative cut-off of 0.75 to determine whether the study is considered of good quality.^20^

## Results

### Study selection

Of 7,465 articles found, 19 met the eligibility criteria. We added 3 articles using reference lists of relevant reviews and articles, resulting in a total of 22 articles included in the review (Figure 1).

### Quality assessment

QualSyst scores ranged from 0.82 to 1 (Table S2). Bias was mostly found in not considering confounding variables or not displaying the estimated variances of the results. As we used a relatively conservative cut-off of 0.75, all 22 studies were considered to have good quality.

### Study characteristics

Of the 22 included studies, 21 had a quantitative research design and 1 had a mixed-method design.^21^ Every study had a prospective longitudinal cohort design, except for 2 in which a secondary analysis of prospectively gathered data was conducted.^22^^,^^23^

The mean age of the population in the studies reporting mean age was 73.7 years, and the median age in the studies reporting median age was 76.2 years. Seventeen studies had both men and women in the cohort.^10^^,^^13^^,^^21^^,^^22^^,^^24–41^ Two studies included female breast cancer patients only,^42^^,^^43^ and 4 studies included male prostate cancer patients only.^23^^,^^44–46^ Other studies included only people with colorectal cancer (*n* = 2),^26^^,^^33^ colon cancer (*n* = 1),^34^ thoracic cancer (*n* = 1),^41^ multiple myeloma (*n* = 1),^22^ all cancer types (*n* = 4),^21^^,^^24^^,^^27^^,^^36^ solid tumors (*n* = 3),^13^^,^^28^^,^^40^ or multiple cancer types (*n* = 4).^29–31^^,^^38^ Most studies included people with all cancer stages (*n* = 15),^13^^,^^21^^,^^23^^,^^24^^,^^27–31^^,^^33^^,^^36^^,^^38^^,^^40^^,^^41^^,^^45^ 4 included only people with non-metastatic cancers (stage I-III),^22^^,^^26^^,^^43^^,^^44^ 2 included only people with metastatic cancers,^42^^,^^46^ and 1 included people with cancer stages II and III^34^ (Table 1).

**Table 1.** Overview of the study and population characteristics.

Study characteristics						Population characteristics			
Author; Year	Country	Main objectives of the study	Study design	Sample size	Within subject analysis?	Age limit + Mean age OR median age	Gender	Cancer type	Cancer stage
**Baziliansky et al., 2023**	13 European countries	To investigate trajectories of depression and QoL over time among cancer survivors compared to individuals without cancer and identify associated factors	Quantitative Longitudinal trajectory study	1066	Yes Group based trajectory model + linear mixed model	≥65 years Mean age of 66,14	Both	All	All
**Cummings et al., 2022**	UK	To identify those older people most at risk of poor QoL and health status in the five years following CRC treatment	Quantitative Longitudinal cohort study	501	Yes Repeated measures + between group analyses	≥65 years Mean age of 68	Both	Colorectal cancer	Non-metastatic
**De Boer et al., 2020**	The Netherlands	To assess the prevalence of psychosocial problems in older patients with metastatic breast cancer, and to assess longitudinal changes in functional status, psychosocial functioning, and QoL	Quantitative Prospective cohort study	85	Yes Linear mixed model	≥70 years Median age of 77	Women	Breast cancer	Metastatic
**Decoster et al., 2018**	Belgium	To investigate HRQoL life at baseline and at follow-up in older patients with cancer and to determine prognostic factors for HRQoL decline	Quantitative Prospective observational cohort study	2971	No Focus on subgroup analyses not individuals	≥70 years Median age of 79	Both	Solid tumor	All
**Esbensen et al., 2007**	Denmark	To investigate possible changes in QoL in elderly persons diagnosed with cancer	Quantitative Prospective study	75	Yes Friedman & Wilcoxon’s signed rank test	≥65 years Median age of 75,49	Both	Breast, colorectal, gynecological, lung cancer	All
**Ikander et al., 2021**	Denmark	To evaluate differences in treatment expectations and quality of life between patients with thoracic cancer	Quantitative Prospective longitudinal study	18	Yes One way and repeated measures ANOVA	Included results only from subgroup of ≥ 70 years Median age of 75	Both	Thoracic cancer	All
**Jayadevappa et al., 2006**	USA	To determine the association of ethnicity with post-treatment recovery of HRQoL outcomes and satisfaction with care among African American and Caucasian elderly men with newly diagnosed prostate cancer	Quantitative Observational prospective cohort study	182	Yes ANOVA	≥65 years Mean age of 71,25 (African Americans) and 69,87 (Caucasian)	Men	Prostate cancer	T1N0M0 - T3bN0M0 (= non metastatic)
**Kaufmann et al., 2015**	Germany	To examine general and elderly specific domains of HRQoL during and after radiotherapy	Quantitative Prospective study	50	Yes Wilcoxon signed rank test	≥80 years Median age of 82	Both	All	All
**Kirkhus et al., 2019**	Norway	To identify potentially modiﬁable factors affecting older patients’ physical function and QoL during cancer treatment.	Quantitative Prospective observational study	288	Yes Linear mixed model	≥70 years Mean age of 76,9	Both	Solid tumors	All
**Litwin et al., 2001**	USA	Examined change in HRQoL during the 12 months before death in men with prostate cancer	Quantitative Longitudinal observational cohort study	131	Partially Analyses of trends over time but focus on group trends	Mean age 75,5 years	Men	Prostate cancer	All
**Maurer et al., 2021**	Germany	To investigate breast cancer survivor’s QoL before diagnosis, during treatment, as well as 5 and 10 years after diagnosis	Quantitative Case-control Cohort study	378 (65+)	Yes Paired T-test + Wilcoxon signed rank test + ANOVA	Included results only from subgroup of ≥65. No mean/median reported	Women	Breast cancer	Stages I-IIIa
**Melmed et al., 2002**	USA	To identify the rates of decline in HRQoL during the year before death in men with prostate cancer	Quantitative Longitudinal observational cohort	23	Yes Calculated individual change between 2 timepoints	Aged 67 to 82 with mean age of 73,8	Men	Prostate cancer	Metastatic
**Mian et al., 2020**	USA	To understand the changes that occurred in geriatric domains and QoL parameters as older adults underwent treatment for MM	Quantitative Secondary analysis of prospective cohort study	36	Yes McNemar + Wilcoxon signed rank test	≥65 years Median age 70	Both	Multiple Myeloma	Stage I-III
**Montroni et al., 2022**	Hospitals world-wide	To compare QoL before and after surgery and identify predictors of decline in QoL	Quantitative Prospective observational cohort study	942	Yes McNemar + Linear mixed model	≥70 years Median age of 78	Both	Solid tumors	All
**Pivodic et al., 2021**	Belgium and The Netherlands	To examine changes in physical, psychological, and social wellbeing in the last 5 years of life of older people with cancer	Quantitative Prospective cohort study	107	Yes Linear mixed model	≥70 years Mean age of 77	Both	Breast, prostate, lung, or gastrointestinal cancer	All
**Posielski et al., 2021**	USA	To investigate impact of age and race on HRQoL in men undergoing radical prostatectomy for localized prostate cancer	Quantitative Secondary analysis of prospectively collected data	626, (57 70+)	Yes, Generalized Estimating Equations (GEE) and repeated measures	Included results only from subgroup of >70. No mean/median reported	Men	Prostate cancer	All
**Puts et al., 2011**	Canada	To report the quality of life of older cancer patients during the first year after diagnosis and factors influencing QoL	Quantitative Prospective cohort study	112	Yes ANOVA	≥65 years Median age of 74,1	Both	Breast, colorectal, lung and lymphoma, myeloma	All
**Reeve et al., 2009**	USA	To quantify the nature and extent of HRQoL changes from before to after cancer diagnosis for 9 types of cancer patients and to compare their health with individuals without cancer	Quantitative Prospective population-based study	1432	Yes ANCOVA	≥65 years Mean age of 73,86	Both	Prostate, breast, colorectal, lung, bladder, endometrial, kidney, melanoma or non-Hodgkin lymphoma	All
**Ronning et al., 2016**	Norway	To examine long-term HRQoL in older surgical patients with colorectal cancer	Quantitative Prospective study	180	Yes ANOVA	≥70 years Mean age of 80	Both	Colorectal cancer	All
**Scheepers et al., 2023**	The Netherlands	To evaluate HRQoL trajectory during the first year after cancer treatment in patients with resectable primary colon cancer	Quantitative Prospective observational cohort study	458 (181 70+)	Yes Paired T-test + changes over time	Included results only from subgroup of ≥70. Mean age 75,4	Both	Colon cancer	Stage II-III
**Taylor et al., 2023**	USA	To examine the longitudinal associations and causal relationships between life-space mobility and QoL	Quantitative Longitudinal observational cohort study	153	Yes General linear mixed model + cross-lagged panel model	≥65 years Mean age of 76,1	Both	All	All
**Tolstrup et al., 2023**	Denmark	To determine whether HRQoL findings are truly reflective of cancer disease and treatment, as opposed to external factors	Mixed method Longitudinal prospective cohort study	21	Yes Thematic analyses + mixed method effects linear regression	≥70 years Median age of 74	Both	All	All

*Abbreviations:* CRC, colorectal cancer; HRQoL, health-related quality of life; MM, multiple myeloma; QoL, quality of life; .

### Synthesis of results

#### Conceptualization and operationalization of quality of life

Most studies (19/22) did not provide a definition or use a framework or conceptualization of quality of life or well-being. The study of Cummings et al. (2022) used Foster and Fenlon’s conceptual model of recovery after cancer treatment,^26^ which states that there are 5 domains of well-being: namely, socio-demographic (pre-existing), clinical, treatment, environmental, and personal. The other 2 articles that reported a form of conceptualization did not use a framework yet explained that quality of life is multi-dimensional and is affected by and affects numerous other functions and aspects.^38^^,^^43^

**Figure 2.**
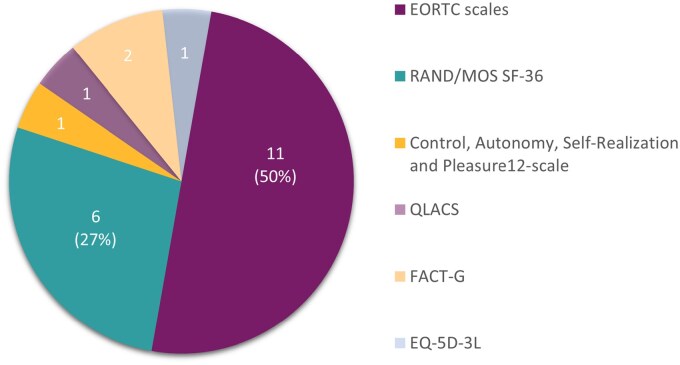
Quality-of-life measurement instruments.

The EORTC QLQ-C30 was most often used to measure quality of life: That is, in 11 studies,^13^^,^^21^^,^^27^^,^^29^^,^^30^^,^^33^^,^^34^^,^^38^^,^^40^^,^^42^^,^^43^ followed by the MOS/RAND Health Survey Short Form^23^^,^^31^^,^^36^^,^^44–46^ in 6 studies (Figure 2). The number of measurement moments in the articles varied between 2 and 13 with an average of 4.4 (median of 3).

#### Changes in overall quality of life

**Figure 3.**
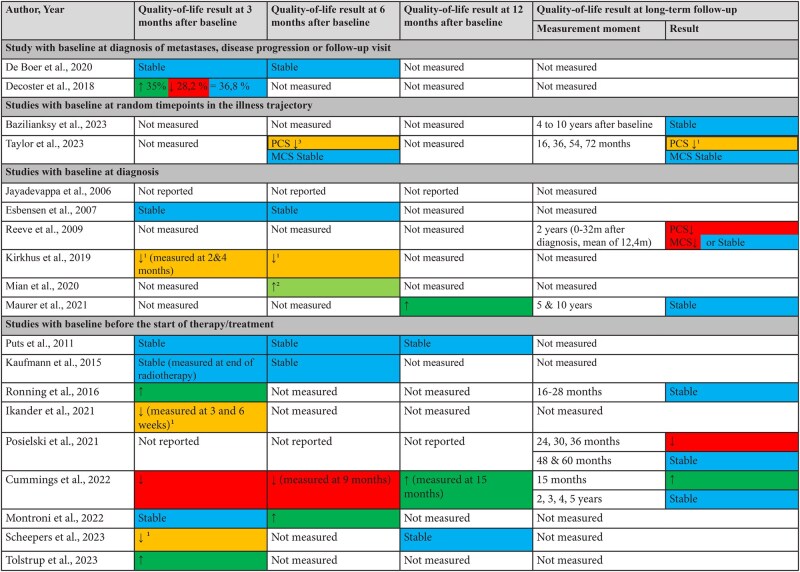
Changes in overall quality of life of older adults with cancer throughout their illness trajectory. ^1^Significant, but not clinically relevantBlue: stable trajectory ^2^Non-significant, but clinically relevantRed: decline in quality of life in comparison to baseline ³Non-significant, no mention of clinical relevanceGreen: increase in quality of life in comparison to baseline Abbreviations: PCS: Physical Component Subscale; MCS: Mental Component Subscale

Six studies measured quality of life starting from diagnosis. One of those reported a stable quality-of-life trajectory,^38^ 1 a non-clinically relevant decline,^13^ 1 a decline after 2 years,^31^ and 2 reported an improvement in quality-of-life scores^22^^,^^43^ (Figure 3). Nine studies measured quality of life starting at the start of a treatment or before surgery, with only Cummings et al. (2022) reporting a decline in quality-of-life scores at 3 and 9 months after the start of the treatment.^26^

Five studies measured quality of life using the EORTC-QLQ-C30 and reported on 1 or multiple sub-scales. Three studies reported a decrease in emotional functioning, whereas other sub-scales mostly remained stable. Four studies used the RAND/MOS SF scale and reported on sub-domains (Table S3).

Nine studies collected long-term follow-up data, that is, 12 months or more after baseline, with baseline occurring at different time points in the disease trajectory. Eight of them^23^^,^^24^^,^^26^^,^^30^^,^^33^^,^^34^^,^^36^^,^^43^ reported quality-of-life scores that were equal to baseline at the final measurement moment, except for Reeve et al. (2009), who found a statistically greater decline in the Physical Component Subscale scores of the SF-36 quality-of-life questionnaire from baseline to follow-up (2 years later) for cancer patients compared to healthy controls. The Mental Component Subscale score only decreased significantly for lung, colorectal, and prostate cancer patients^31^.

Cummings et al. (2022), Maurer et al. (2021), and Scheepers et al. (2023) found lower quality of life during and shortly after treatment, followed by increasing quality-of-life scores, resulting in similar scores to those at baseline. Ikander et al. (2021) also reported a declining quality-of-life trend during treatment but did not measure at a long-term follow-up.

In the 1 study with a mixed-method design, participants reported through qualitative interviews conducted 3 months after starting the treatment: (1) feeling more at ease, less anxious, and less worried about the future compared to the beginning of treatment, (2) experiencing more fatigue than before the cancer diagnosis but still feeling satisfied with their circumstances, (3) noting that the influence of comorbidity at baseline remained significant at follow-up, and (4) acknowledging several side effects but feeling they were gradually regaining their pre-diagnosis quality of life.^21^ The quantitative survey in this study showed a significant and clinically relevant improvement in quality of life, except among people receiving palliative treatment. Combining the results from interviews and surveys, the authors concluded that receiving a cancer diagnosis significantly reduced overall quality of life among older adults, but substantial improvement was observed 3 months after starting treatment.^21^

A detailed overview of all results on changes in quality of life of older adults with cancer can be found in Table S4.

Changes in quality of life in the last years of life

Three studies examined changes in quality of life in the last years of life, and all of them found declining trends^29^^,^^45^^,^^46^ (Table 2). Pivodic et al. (2021) did not measure overall quality of life but found a decline in several sub-domains of quality of life over the last 5 years of life.^29^

**Table 2.** Quality-of-life changes in last years of life.

Author, year	Instrument	Measure moments	Changes in overall QoL	Changes in quality-of-life subdomains
Litwin et al., 2001^45^	RAND SF-36 (Difference from 6 to 8 points in considered clinically relevant)	3 months interval in last year of life	Significant decrease.	Significant decrease in all 8 SF-36 domains in last 12 months of life. Most substantial domains of decline: Role limitations due to physical problems (29 points) Emotional problems (26 points) Physical problems (24 points) Social function (23 points) Bodily pain (18 points). Smallest decrease: wellbeing (10 points). Significant decrease in role limitations due to emotional problems and energy fatigue.
Melmed et al., 2002^46^	RAND SF-36 (Difference from 6 to 8 points in considered clinically relevant)	3 months interval in last year of life	HRQoL (both PCS and MCS) declined, but this was non-significant	Different subgroups (wellbeing in married patients, general health perceptions, social functioning, and emotional wellbeing in those with at least a college degree and role functioning due to emotional functioning, social functioning, and general health perceptions in those with annual household incomes over 30,000 dollars) had significant decline in QoL. Affluent patients had worse declines in MCS (significant), while lower income patients’ trend toward lower PCS (non-significant). PCS had a slower decline in married men, and better in men with higher incomes (non-significant). MCS was worse in men with higher incomes.
Pivodic et al., 2021^29^	Physical, social, role, emotional functioning of EORTC QLQ C30	At diagnosis and 6 months, 1, 3, and 5 years later	No results on overall QoL	Physical functioning and role functioning declined. Emotional functioning and social functioning declined slightly and non-significant. Also, a small and non-significant decline in emotional and social functioning.

*Abbreviations:* MCS, Mental Component Subscale; PCS, Physical Component Subscale; QoL, quality of life.

#### Factors associated with changes in quality of life

**Figure 4.**
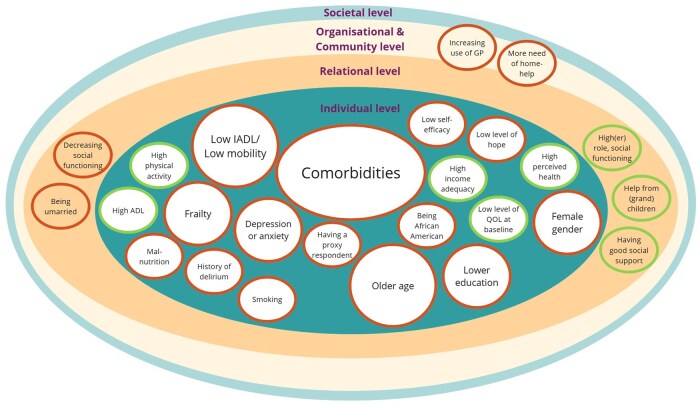
Factors associated with changes in quality of life. Abbreviations: Red = negative association with QoLQoL: Quality of life; Green = positive association with QoLGP: General Practitioner; Bigger spheres = mentioned in more studies(I)ADL: (Instrumental) Activities of Daily Living

Individual-level factors associated with quality of life were studied in 10 studies^10^^,^^13^^,^^21^^,^^23^^,^^24^^,^^26^^,^^28^^,^^31^^,^^38^^,^^44^; 4 studies examined relational factors^24^^,^^26^^,^^31^^,^^38^; and 1 study mentioned community-level factors.^38^ No study examined society-level factors associated with changes in quality of life (Figure 4).

The individual-level factors associated with declining quality of life encompass 2 categories: (1) physical factors such as having comorbidities or low mobility and (2) mental factors such as depression or low level of self-efficacy to manage illness-related problems. Having comorbidities,^13^^,^^21^^,^  ^26^^,^^31^ older age,^23^^,^^24^^,^^26^ and having problems with mobility and/or daily tasks^13^^,^^31^^,^^38^ were most frequently mentioned. At the relational level, being unmarried, and experiencing decreasing social functioning were associated with a decline in quality of life,^24^^,^^38^ whereas good social support, strong social functioning, and help from children or grandchildren were associated with quality-of-life improvements.^26^^,^^31^^,^^38^ The 1 study examining community-level factors found that a greater need for home-help services, district nurses, and increased use of general practitioners were associated with a decline in quality of life.^38^

## Discussion and conclusion

This systematic review synthesized the findings of 22 studies examining changes in quality of life among older adults with various cancer types and stages and the factors associated with these changes. We only found quantitative studies except for 1 mixed-method study. Most studies lacked an explicit definition or conceptualization of quality of life or well-being. Rather than a single trajectory, this review highlights multiple potential quality-of-life trends. Three studies examining quality of life in the last years of life consistently reported a decline toward the end of life. In contrast, among the 19 studies not focused on the end of life, declines in quality of life were less common. Five studies observed a decline during treatment, which in 4 studies was followed by improvements afterwards, with quality of life returning to or exceeding baseline levels. Of the 9 studies examining changes in quality of life 12 months or more after baseline, 8 found stable scores at the final follow-up, while 1 found a significant and clinically relevant decline. Key individual-level factors associated with a declining quality of life were physical factors, such as having comorbidities, and mental factors, such as depression. Remarkably, relational, community, and societal factors were rarely studied, highlighting critical knowledge gaps.

### Strengths and limitations

The strengths of this review include the systematic search in 3 databases, manual searches of reference lists from relevant articles and reviews, and collaboration among multiple researchers for data screening and extraction, ensuring high methodological quality. One limitation is that we excluded studies with only a single measurement moment, potentially overlooking retrospective qualitative studies that explored changes over time. Also, some studies measuring quality of life or well-being may have been missed if they only used alternative terms such as general health, life satisfaction, or wellness, which were excluded during our screening.

### Changes in quality of life throughout the illness trajectory of older adults with cancer

Although the studies varied in the timing of the measurements and in cancer populations being studied (ie, types and illness stages), we were able to identify trends in quality-of-life changes. First, we found 3 studies examining quality of life in the last year of life, all reporting a declining trend. Research on quality of life at the end of life in older adults with cancer is scarce, yet studies on quality of life of adults across all ages with cancer also concluded that quality-of-life declines in the last year of life, especially in the last 3 months of life.^47^^,^^48^ Second, among the studies in this review that did not focus on the end of life but included a follow-up of 12 months or longer (*n* = 9), quality of life was generally stable or even improved compared to baseline. These findings contrast with previous research across all ages, which suggests that advancing cancer and functional decline typically lead to a deterioration in quality of life, particularly in older adults.^49^^,^^50^ However, a recent study does concur with our findings, reporting that changes in quality of life in palliative care are common, with trajectories varying both within and between individuals, and they do not always, or even frequently, follow a downward trend.^51^ Third, 4 studies in this review found an initial decline in quality of life due to treatment or surgery, followed by improvements to levels similar or even higher than before treatment.^21^^,^^26^^,^^34^^,^^43^ This aligns with findings from a systematic review by Hohls et al. (2021) that, while quality of life declines during serious illness, it often increases with remission, returning to or exceeding pre-disease levels.^52^

### Factors associated with changes in quality of life

In line with other reviews,^53^^,^^54^ most studies in this review focused on physical individual-level factors associated with changes in quality of life, such as having comorbidities, age and problems with daily tasks. Some studies also examined relational factors, such as the impact of being married or needing help, but a significant knowledge gap remains in this area. Only 2 community-level and no societal-level factors were identified in the studies included in this review, likely because such factors might be easier to capture through qualitative research. Traditionally, quality of life in medicine and health science has been measured quantitatively, though there is growing recognition of the importance of qualitative approaches in understanding quality-of-life dynamics.^4^

### Conceptualization of quality of life and well-being

Many studies in this review lacked clear definitions and conceptualizations of quality of life and well-being. Only 3 studies^26^^,^^38^^,^^43^ explicitly defined quality of life or well-being, and, even then, they were used interchangeably. The study of Cummings et al. (2022), for example, explains Foster and Fenlon’s conceptual model of well-being but proceeds with a measurement instrument measuring quality of life. Pivodic et al. (2021) used sub-scales of EORTC QLQ C-30 to measure changes in well-being, while others use these for quality of life. Moreover, the study of Baziliansky et al. (2023) uses the Control, Autonomy, Self-Realization, and Pleasure scale to measure quality of life, whereas the concepts of autonomy and self-realization are fundamentals of eudaimonic well-being.^55^ Remarkably, none of the studies used the WHO definition or conceptual frameworks of well-being or quality of life.

### Implications for practice and future research

Clinicians caring for older adults with cancer should be aware that, while some experience changes in their quality of life, this is not the case for all older adults, as we found several longitudinal studies showing stable trajectories. Periods that merit attention include treatment and end-of-life periods, both found to be related to declines in quality of life or well-being. Support during these periods seems particularly crucial. Subsequently, in interventions and trials, researchers should think carefully when setting a feasible goal, depending on the timing of their intervention. Qualitative or mixed-methods designs to capture nuanced trajectories of quality of life should be incorporated. This would also enable the identification of relational, community, and societal factors associated with changes in quality of life and well-being in older adults with cancer. This knowledge will help researchers to target interventions and will make clinicians aware of what might affect quality of life. The methodological heterogeneity across studies in this review, combined with the relatively small number of studies examining quality-of-life trajectories in older adults with cancer, made comparison of results challenging. Future research should focus on generating more evidence on quality of life and well-being in this population, including studies focusing on specific aspects or sub-domains of quality of life, with an emphasis on methodological consistency and long-term follow-up in heterogeneous populations. Furthermore, studies comparing quality-of-life trajectories of older people with cancer with younger people with cancer, would be informative for both clinicians and researchers in choosing fitting interventions, care or treatment. A unified framework for defining and measuring quality of life and well-being is currently lacking, but it is crucial to enhance the comparability and utility of research findings.

### Conclusion

Despite the methodological heterogeneity between studies, we identified trends in quality-of-life changes across the illness trajectories of older adults with cancer, particularly declining trends during treatment and end-of-life periods and stable trends in long-term follow-up. Factors associated with changes in quality of life were primarily identified on the individual and relational levels.

## Supplementary Material

oyaf223_Supplementary_Data

## Data Availability

All data are available in the appendices of this article or upon request from the corresponding author (CV). This includes the search string, the data extracted and analyzed, and the quality assessments of the included studies.
