# Supplementary material for: Changes in quality of life throughout the illness trajectory of older adults with cancer: a systematic review
Source: Oncologist. 2025 Jul 23;30(9):oyaf223. doi: 10.1093/oncolo/oyaf223 (PMC12445657; doi:10.1093/oncolo/oyaf223)
Supplement: oyaf223_Supplementary_Data [file oyaf223_supplementary_data.docx]

**Supplementary table 1. Search strategy**

*Search string PubMed*

| Older people | ((("Aged, 80 and over"[MeSh] OR "Aged, 80 and over"[tiab] OR "olde*"[tiab] OR "very old"[tiab] OR "nonagenarian*"[tiab] OR "octogenarian*"[tiab] OR "centenarian*"[tiab] OR "supercentenarian*"[tiab] OR "aged"[mesh] OR "aged"[tiab] OR "elder*"[tiab] OR "geriatric"[tiab] OR "ageing"[tiab] OR "aging"[tiab] OR "old people"[tiab] OR "end of life"[tiab] OR "last year of life"[tiab] OR “last years of life”[tiab] OR "end-of-life"[tiab] NOT "caregivers"[mesh]) |  |
| --- | --- | --- |
| Cancer | AND ("neoplasms"[mesh] OR "neoplasm*"[tiab] OR "oncolog*"[tiab] OR "cancer*"[tiab] OR "neoplas*"[tiab] OR "tumour*"[tiab] OR "tumor*"[tiab] OR "carcinom*"[tiab] OR "melanom*"[tiab] OR "lymphom*"[tiab] OR "leukemi*"[tiab] OR "malignan*"[tiab] OR "metasta*"[tiab] OR "carcinogen*"[tiab] OR "oncogen*"[tiab] OR "anticarcinogen*"[tiab] OR "sarcoma*"[tiab] OR "precancerous"[tiab] OR "paraneoplastic"[tiab] OR "neuroma*"[tiab] OR "blastoma*"[tiab] OR "meningioma*"[tiab] OR "lymphangioma*"[tiab] OR "lymphangiomyoma*"[tiab] OR "lymphangiosarcoma*"[tiab] OR "hodgkin disease"[tiab] OR "plasmacytoma*"[tiab] OR "carcinosarcoma*"[tiab] OR "hepatoblastoma*"[tiab] OR "mesenchymoma*"[tiab] OR "chordoma*"[tiab] OR "germinoma*"[tiab] OR "gonadoblastoma*"[tiab] OR "mesonephroma*"[tiab] OR "teratoma*"[tiab] OR "teratocarcinoma*"[tiab] OR "nsclc"[tiab]) |  |
| Quality of life | AND ("overall health"[tiab] OR "general health"[tiab] OR "health level*"[tiab] OR "quality of life"[mesh] OR "quality of life"[tiab] OR "life qualit*"[tiab] OR "living qualit*"[tiab] OR "Health-Related Quality of life"[tiab] OR "Health Related Quality of Life"[tiab] OR "HRQOL"[tiab] OR "hrql"[tiab] OR "qol"[tiab] OR "well-being"[tiab] OR "wellness"[tiab] OR "psychological well being"[tiab] OR "Wellbeing"[tiab] OR "psychological Ill being"[tiab] OR "life satisfaction*"[tiab]) |  |
| Longitudinal studies | AND (“Longitudinal studies”[mesh] OR “longitudinal stud*”[tiab] OR “longitudinal survey*”[tiab] OR “longitudinal*”[tiab] OR “trajectory”[tiab] OR “trajectories”[tiab] OR “illness trajectory*”[tiab] OR “follow-up studies”[mesh] OR “follow-up stud*”[tiab] OR “followup stud*”[tiab] OR "prospective studies"[mesh] OR "prospective stud*"[tiab] NOT ("cross-sectional studies"[mesh] OR "cross-sectional stud*"[tiab] OR "cross-sectional survey*"[tiab]))) |  |
| NOT Clinical trials | NOT ("randomized controlled trial"[pt] OR "controlled clinical trial"[pt] OR "randomized controlled trials as topic"[mh] OR "random allocation"[mh] OR "double-blind method"[mh] OR "single-blind method"[mh] OR "clinical trial"[pt] OR "clinical trials as topic"[mh] OR "clinical trial"[tw] OR ((singl*[tw] OR doubl*[tw] OR trebl*[tw] OR tripl*[tw]) AND (mask*[tw] OR blind*[tw])) OR "latin square"[tw] OR "placebos"[mh] OR "placebo*"[tw] OR "random*"[tw] OR "research design"[mh:noexp] OR "comparative study"[pt] OR "evaluation studies"[pt] OR "evaluation studies as topic"[mesh] OR "evaluation stud*"[tiab] OR "treatment outcome"[mesh] OR "treatment outcome*"[tiab]) |  |
| NOT Validation studies | NOT ("validation studies as topic"[mesh] OR “validation study”[pt] OR "validation stud*"[tiab] OR “feasibility stud*”[tiab] OR “pilot stud*”[tiab]) |  |
| NOT Child | NOT ("Adolescent"[Mesh] OR "Child"[Mesh] OR "Infant"[Mesh] OR "adolescen*"[tiab] OR "child*"[tiab] OR "schoolchild*"[tiab] OR "infant*"[tiab] OR "girl*"[tiab] OR "boy"[tiab] OR "boys"[tiab] OR "teen"[tiab] OR "teens"[tiab] OR "teenager*"[tiab] OR "youth*"[tiab] OR "pediatr*"[tiab] OR "paediatr*"[tiab] OR "puber*"[tiab] OR "birth cohort"[mesh])) |  |
| 1 AND 2 AND 3 AND 4 NOT 5 NOT 6 NOT 7 |  | **3.914** |

*Search string Embase*

| Older people | (((‘senescence’/exp OR ‘senescence’:ti,ab,kw OR ‘Aged, 80 and over’:ti,ab,kw OR ‘olde*’:ti,ab,kw OR ‘very old’:ti,ab,kw OR ‘nonagenarian*’:ti,ab,kw OR ‘octogenarian*’:ti,ab,kw OR ‘centenarian*’:ti,ab,kw OR ‘supercentenarian*’:ti,ab,kw OR ‘very elderly’/exp OR ‘aged’:ti,ab,kw OR ‘very elderly’:ti,ab,kw OR ‘elder*’:ti,ab,kw OR ‘geriatric’:ti,ab,kw OR ‘ageing’:ti,ab,kw OR ‘aging’:ti,ab,kw OR ‘old people’:ti,ab,kw OR ‘end of life’:ti,ab,kw OR ‘last year of life’:ti,ab,kw OR ‘end-of-life’:ti,ab,kw OR ‘last years of life’:ti,ab,kw NOT ‘caregiver’/exp) |  |
| --- | --- | --- |
| Cancer | AND (‘neoplasm’/exp OR ‘neoplasm*’:ti,ab,kw OR ‘oncolog*’:ti,ab,kw OR ‘cancer*’:ti,ab,kw OR ‘neoplas*’:ti,ab,kw OR ‘tumour*’:ti,ab,kw OR ‘tumor*’:ti,ab,kw OR ‘carcinom*’:ti,ab,kw OR ‘melanom*’:ti,ab,kw OR ‘lymphom*’:ti,ab,kw OR ‘leukemi*’:ti,ab,kw OR ‘malignan*’:ti,ab,kw OR ‘metasta*’:ti,ab,kw OR ‘carcinogen*’:ti,ab,kw OR ‘oncogen*’:ti,ab,kw OR ‘anticarcinogen*’:ti,ab,kw OR ‘sarcoma*’:ti,ab,kw OR ‘precancerous’:ti,ab,kw OR ‘paraneoplastic’:ti,ab,kw OR ‘neuroma*’:ti,ab,kw OR ‘blastoma*’:ti,ab,kw OR ‘meningioma*’:ti,ab,kw OR ‘lymphangioma*’:ti,ab,kw OR ‘lymphangiomyoma*’:ti,ab,kw OR ‘lymphangiosarcoma*’:ti,ab,kw OR ‘hodgkin disease’:ti,ab,kw OR ‘plasmacytoma*’:ti,ab,kw OR ‘carcinosarcoma*’:ti,ab,kw OR ‘hepatoblastoma*’:ti,ab,kw OR ‘mesenchymoma*’:ti,ab,kw OR ‘chordoma*’:ti,ab,kw OR ‘germinoma*’:ti,ab,kw OR ‘gonadoblastoma*’:ti,ab,kw OR ‘mesonephroma*’:ti,ab,kw OR ‘teratoma*’:ti,ab,kw OR ‘teratocarcinoma*’:ti,ab,kw OR ‘nsclc’:ti,ab,kw) |  |
| Quality of life | AND (‘overall health’:ti,ab,kw OR ‘general health’:ti,ab,kw OR ‘health level*’:ti,ab,kw OR ‘quality of life’/exp OR ‘quality of life’:ti,ab,kw OR ‘life qualit*’:ti,ab,kw OR ‘living qualit*’:ti,ab,kw OR ‘Health-Related Quality of life’:ti,ab,kw OR ‘Health Related Quality of Life’:ti,ab,kw OR ‘HRQOL’:ti,ab,kw OR ‘hrql’:ti,ab,kw OR ‘qol’:ti,ab,kw OR ‘well-being’:ti,ab,kw OR ‘wellness’:ti,ab,kw OR ‘wellbeing’/exp OR ‘psychological well being’:ti,ab,kw OR ‘Wellbeing’:ti,ab,kw OR ‘psychological Ill being’:ti,ab,kw OR ‘life satisfaction’:ti,ab,kw) |  |
| Longitudinal study | AND (‘Longitudinal studies’:ti,ab,kw OR ‘longitudinal study’/exp OR ‘longitudinal study’:ti,ab,kw OR ‘longitudinal survey*’:ti,ab,kw OR ‘longitudinal*’:ti,ab,kw OR ‘trajectory’:ti,ab,kw OR ‘trajectories’:ti,ab,kw OR ‘illness trajectory*’:ti,ab,kw OR 'prospective study'/exp OR 'prospective stud*':ti,ab,kw OR ‘follow-up’/exp OR ‘follow-up’:ti,ab,kw OR ‘followup stud*’:ti,ab,kw OR ‘follow up stud*’:ti,ab,kw NOT (‘cross-sectional study’/exp OR ‘cross-sectional stud*’:ti,ab,kw))) |  |
| NOT Validation study | NOT (‘validation study’/exp OR ‘validation stud*’:ti,ab,kw OR ‘feasibility study’/exp OR ‘feasibility stud*’:ti,ab,kw OR ‘pilot study’/exp OR ‘pilot stud*’:ti,ab,kw) |  |
| NOT child | NOT ('adolescent'/exp OR 'child'/exp OR ‘adolescent*’:ti,ab,kw,kw OR ‘child*’:ti,ab,kw OR ‘schoolchild*’:ti,ab,kw OR ‘infant*’:ti,ab,kw OR ‘girl*’:ti,ab,kw OR ‘boy*’:ti,ab,kw OR ‘teen*’:ti,ab,kw OR ‘teenager*’:ti,ab,kw OR ‘youth*’:ti,ab,kw OR ‘pediatr*’:ti,ab,kw OR ‘paediatr*’:ti,ab,kw OR ‘puber*’:ti,ab,kw OR ‘birth cohort’/exp OR ‘embryo’/exp OR ‘fetus’/exp OR ‘juvenile’/exp) |  |
| NOT clinical trials | NOT ('clinical trial'/exp OR 'triple blind procedure'/exp OR 'double blind procedure'/exp OR 'single blind procedure'/exp OR ‘controlled study’/exp OR 'randomization'/exp OR 'placebo'/exp OR 'methodology'/de OR 'comparative study'/de OR 'evaluation study'/de OR 'crossover procedure'/exp OR 'clinical trial':ab,ti OR ((singl*:ab,ti OR doubl*:ab,ti OR trebl*:ab,ti OR tripl*:ab,ti) AND (mask*:ab,ti OR blind*:ab,ti)) OR 'latin square':ab,ti OR placebo*:ab,ti OR random*:ab,ti OR control:ab,ti OR controll*:ab,ti)) |  |
| 1 AND 2 AND 3 AND 4 NOT 5 NOT 6 NOT 7 |  | 1 509 |

*Search string PsycINFO*

| Older people | (((MAINSUBJECT.EXACT.EXPLODE(“older adulthood”) OR MAINSUBJECT.EXACT.EXPLODE(“aging”) OR TI,AB,SU(“older adulthood”) OR TI,AB,SU(“aging”) OR TI,AB,SU(“aged, 80 and over”) TI,AB,SU(“aged, 65 and over”) OR TI,AB,SU(“olde*”) OR TI,AB,SU(“very old”) OR TI,AB,SU(“nonagenarian*”) OR TI,AB,SU(“octogenarian*”) OR TI,AB,SU(“centenarian*”) OR TI,AB,SU(“supercentenarian*”) OR TI,AB,SU(“aged”) OR MAINSUBJECT.EXACT.EXPLODE(“aged (attitudes toward)”) OR TI,AB,SU(“elder*”) OR MAINSUBJECT.EXACT.EXPLODE(“geriatric patients”) OR TI,AB,SU(“geriatric”) OR TI,AB,SU(“end of life”) OR TI,AB,SU(“end-of-life”) OR TI,AB,SU(“last years of life”) OR TI,AB,SU(“last year of life”) OR TI,AB,SU(“ageing”) OR TI,AB,SU(“aging”) OR TI,AB,SU(“old people”) NOT MAINSUBJECT.EXACT(“caregivers”)) |  |
| --- | --- | --- |
| Cancer | AND (MAINSUBJECT.EXACT.EXPLODE(“neoplasms”) OR TI,AB,SU(“neoplasm*”) OR TI,AB,SU(“cancer*”) OR MAINSUBJECT.EXACT.EXPLODE(“oncology”) OR TI,AB,SU(“oncolog*”) OR TI,AB,SU(“neoplas*”) OR TI,AB,SU(“tumour*”) OR TI,AB,SU(“tumor*”) OR TI,AB,SU(“carcinom*”) OR TI,AB,SU(“melanom*”) OR TI,AB,SU(“lymphom*”) OR TI,AB,SU(“leukemi*”) OR TI,AB,SU(“malignan*”) OR TI,AB,SU(“metasta*”) OR TI,AB,SU(“carcinogen*”) OR TI,AB,SU(“oncogen*”) OR TI,AB,SU(“anticarcinogen*”) OR TI,AB,SU(“sarcoma*”) OR TI,AB,SU(“precancerous”) OR TI,AB,SU(“paraneoplastic”) OR TI,AB,SU(“neuroma*”) OR TI,AB,SU(“blastoma*”) OR TI,AB,SU(“meningioma*”) OR TI,AB,SU(“lymphangioma*”) OR TI,AB,SU(“lymphangiomyoma*”) OR TI,AB,SU(“lymphangiosarcoma*”) OR TI,AB,SU("hodgkin disease") OR TI,AB,SU(“plasmacytoma*”) OR TI,AB,SU(“carcinosarcoma*”) OR TI,AB,SU(“hepatoblastoma*”) OR TI,AB,SU(“mesenchymoma*”) OR TI,AB,SU(“chordoma*”) OR TI,AB,SU(“germinoma*”) OR TI,AB,SU(“gonadoblastoma*”) OR TI,AB,SU(“mesonephroma*”) OR TI,AB,SU(“teratoma*”) OR TI,AB,SU(“teratocarcinoma*”) OR TI,AB,SU(“nsclc”)) |  |
| Quality of life | AND (TI,AB,SU(“overall health”) OR TI,AB,SU(“general health”) OR TI,AB,SU(“health level*”) OR TI,AB,SU(“quality of life”) OR TI,AB,SU(“life qualit*”) OR TI,AB,SU(“living qualit*”) OR TI,AB,SU(“Health-Related Quality of life”) OR TI,AB,SU(“Health Related Quality of Life”) OR TI,AB,SU(“HRQOL”) OR TI,AB,SU(“hrql”) OR TI,AB,SU(“qol”) OR MAINSUBJECT.EXACT.EXPLODE(“Well Being”) OR TI,AB,SU(“well-being”) OR TI,AB,SU(“wellness”) OR TI,AB,SU(“psychological well being”) OR TI,AB,SU(“Wellbeing”) OR TI,AB,SU(“psychological Ill being”) OR MAINSUBJECT.EXACT(“life satisfaction”) OR TI,AB,SU(“life satisfaction”)) |  |
| Longitudinal | AND (MAINSUBJECT.EXACT.EXPLODE(“Longitudinal Studies”) OR TI,AB,SU(“longitudinal stud*) OR TI,AB,SU(“longitudinal survey*”) OR TI,AB,SU(“trajectory”) OR TI,AB,SU(“trajectories”) OR TI,AB,SU(“illness trajector*”) OR MAINSUBJECT.EXACT.EXPLODE(“followup studies”) OR TI,AB,SU(“followup stud*) OR TI,AB,SU(“follow up stud*”) OR TI,AB,SU(“follow-up stud*”) OR MAINSUBJECT.EXACT.EXPLODE(“Prospective studies”) OR TI,AB,SU(“prospective stud*”) NOT (TI,AB,SU(“cross sectional stud*”) OR TI,AB,SU(“cross-sectional stud*”) OR TI,AB,SU(“cross-sectional survey”)))) |  |
| NOT clinical trials | NOT (MAINSUBJECT.EXACT.EXPLODE(“randomized controlled trials”) OR MAINSUBJECT.EXACT.EXPLODE(“randomized clinical trials”) OR MAINSUBJECT.EXACT.EXPLODE(“clinical trials”) OR MAINSUBJECT.EXACT.EXPLODE(“single-case experimental design”) OR MAINSUBJECT.EXACT.EXPLODE(“between groups design”) OR MAINSUBJECT.EXACT.EXPLODE(“hypothesis testing”) OR MAINSUBJECT.EXACT.EXPLODE(“treatment effectiveness evaluation”) OR TI,AB,SU(“evaluation stud*”) OR MAINSUBJECT.EXACT.EXPLODE(“treatment outcomes”) OR TI,AB,SU(“treatment outcome*”) OR TI,AB,SU(“placebo*”) OR TI,AB,SU(“randomly”) OR TI,AB,SU(“randomi*”) OR ((TI,AB,SU("singl*") OR TI,AB,SU("doubl*") OR TI,AB,SU("trebl*") OR TI,AB,SU("tripl*")) N/3 (TI,AB,SU("blind*") OR TI,AB,SU("mask*") OR TI,AB,SU("dummy"))) OR ((TI,AB(“control*”)) N/3 (TI,AB(“trial*”) OR TI,AB(“study”) OR TI,AB(“studies”) OR TI,AB(“group*”))) OR TI,AB,SU(“allocat*”) OR TI,AB,SU(“assign*”) OR TI,AB,SU(“crossover*”) OR TI,AB,SU(“cross over*”)) |  |
| NOT Validation study | NOT (MAINSUBJECT.EXACT.EXPLODE(“statistical validity”) OR MAINSUBJECT.EXACT.EXPLODE(“test validity”) OR TI,AB,SU(“validation stud*”) OR TI,AB,SU(“feasibility stud*”) OR TI,AB,SU(“pilot stud*”)) |  |
| NOT Child | NOT (MAINSUBJECT.EXACT.EXPLODE(“early adolescence”) OR MAINSUBJECT.EXACT.EXPLODE(“emerging adulthood”) OR MAINSUBJECT.EXACT.EXPLODE(“late adolescence”) OR MAINSUBJECT.EXACT.EXPLODE(“neonatal period”) OR MAINSUBJECT.EXACT.EXPLODE(“perinatal period”) OR MAINSUBJECT.EXACT.EXPLODE(“postnatal period”) OR MAINSUBJECT.EXACT.EXPLODE(“prenatal developmental stages”) OR MAINSUBJECT.EXACT.EXPLODE(“puberty”) OR AGE("adolescence (13-17 yrs)") OR AGE("childhood (birth-12 yrs)") OR AGE("infancy (2-23 mo)”) OR AGE("neonatal (birth-1 mo)") OR AGE("preschool age (2-5 yrs)") OR AGE("school age (6-12 yrs)") OR TI,AB,SU(“adolescen*”) OR TI,AB,SU (“child*”) OR TI,AB,SU(“schoolchild*”) OR TI,AB,SU(“infant*”) OR TI,AB,SU(“girl*”) OR TI,AB,SU(“boy*”) OR TI,AB,SU(“teen”) OR TI,AB,SU(“teens”) OR TI,AB,SU(“teenager*”) OR TI,AB,SU(“youth*”) OR TI,AB,SU(“pediatr*”) OR TI,AB,SU(“paediatr*”) OR TI,AB,SU(“puber*”))) |  |
| 1 AND 2 AND 3 AND 4 NOT 5 NOT 6 NOT 7 |  | 1 214 |

**Supplementary table 2. Quality Assessment table**

| **Quality assessment of included articles – QualSyst tool** | | | | | | | | | | | | | | | | | | | | | | |
| --- | --- | --- | --- | --- | --- | --- | --- | --- | --- | --- | --- | --- | --- | --- | --- | --- | --- | --- | --- | --- | --- | --- |
| 1. **Quantitative studies** | | | | | | | | | | | | | | | | | | | | | | |
| Author, year | Objective | Design | | Subject selection | | Subject characteristics | | Random allocation | | Blinding investigators | Blinding subjects | | Outcomes | Sample size | Analysis | | Estimate variance | | Confounding | Results | Conclusion | Score |
| Baziliansky et al., 2023 | 2 | 2 | | 2 | | 2 | | N/A | | N/A | N/A | | 2 | 2 | 2 | | 2 | | 1 | 1 | 2 | 0,91 |
| Cummings et al., 2022 | 2 | 2 | | 2 | | 2 | | N/A | | N/A | N/A | | 2 | 2 | 2 | | 2 | | 2 | 2 | 2 | 1,00 |
| De Boer et al., 2020 | 2 | 2 | | 2 | | 2 | | N/A | | N/A | N/A | | 2 | 2 | 2 | | 1 | | 2 | 2 | 2 | 0,95 |
| Decoster et al., 2018 | 2 | 2 | | 2 | | 2 | | N/A | | N/A | N/A | | 2 | 2 | 2 | | 2 | | 2 | 2 | 1 | 0,95 |
| Esbensen et al., 2007 | 2 | 2 | | 2 | | 2 | | N/A | | N/A | N/A | | 2 | 2 | 2 | | 2 | | 2 | 2 | 2 | 1,00 |
| Ikander et al., 2021 | 2 | 2 | | 2 | | 2 | | N/A | | N/A | N/A | | 2 | 1 | 2 | | 0 | | 1 | 2 | 2 | 0,82 |
| Jayadevappa et al., 2006 | 2 | 2 | | 2 | | 2 | | N/A | | N/A | N/A | | 2 | 2 | 2 | | 2 | | 2 | 2 | 2 | 1,00 |
| Kaufmann et al., 2015 | 2 | 2 | | 2 | | 2 | | N/A | | N/A | N/A | | 2 | 2 | 2 | | 2 | | 1 | 2 | 2 | 0,95 |
| Kirkhus et al., 2019 | 2 | 2 | | 2 | | 2 | | N/A | | N/A | N/A | | 2 | 2 | 2 | | 2 | | 2 | 2 | 2 | 1,00 |
| Litwin et al., 2001 | 2 | 2 | | 2 | | 2 | | N/A | | N/A | N/A | | 2 | 2 | 2 | | 0 | | 2 | 2 | 1 | 0,86 |
| Maurer et al., 2021 | 2 | 2 | | 2 | | 2 | | N/A | | N/A | N/A | | 2 | 2 | 2 | | 2 | | 0 | 2 | 2 | 0,91 |
| Melmed et al., 2002 | 2 | 2 | | 2 | | 2 | | N/A | | N/A | N/A | | 2 | 1 | 2 | | 2 | | 1 | 2 | 1 | 0,86 |
| Mian et al., 2020 | 2 | 2 | | 2 | | 2 | | N/A | | N/A | N/A | | 2 | 1 | 2 | | 2 | | 0 | 2 | 2 | 0,86 |
| Montroni et al., 2022 | 2 | 2 | | 2 | | 2 | | N/A | | N/A | N/A | | 2 | 2 | 2 | | 2 | | 2 | 2 | 1 | 0,95 |
| Pivodic et al., 2021 | 2 | 2 | | 2 | | 2 | | N/A | | N/A | N/A | | 2 | 2 | 2 | | 2 | | 0 | 1 | 1 | 0,82 |
| Posielski et al., 2021 | 2 | 2 | | 2 | | 2 | | N/A | | N/A | N/A | | 2 | 2 | 2 | | 2 | | 2 | 2 | 2 | 1,00 |
| Puts et al., 2011 | 2 | 2 | | 2 | | 2 | | N/A | | N/A | N/A | | 2 | 2 | 2 | | 2 | | 0 | 2 | 2 | 0,91 |
| Reeve et al., 2009 | 2 | 2 | | 2 | | 2 | | N/A | | N/A | N/A | | 2 | 2 | 2 | | 2 | | 2 | 2 | 1 | 0,95 |
| Ronning et al., 2016 | 2 | 2 | | 2 | | 2 | | N/A | | N/A | N/A | | 2 | 2 | 2 | | 2 | | 0 | 2 | 2 | 0,91 |
| Scheepers et al., 2023 | 2 | 2 | | 2 | | 2 | | N/A | | N/A | N/A | | 2 | 2 | 2 | | 2 | | 0 | 2 | 2 | 0,91 |
| Taylor et al., 2023 | 2 | 2 | | 2 | | 2 | | N/A | | N/A | N/A | | 2 | 2 | 2 | | N/A | | 2 | 1 | 2 | 0,86 |
| Tolstrup et al., 2023 | 2 | 2 | | 2 | | 2 | | N/A | | N/A | N/A | | 2 | 2 | 2 | | 2 | | 0 | 2 | 1 | 0,86 |
| 1. **Qualitative studies** | | | | | | | | | | | | | | | | | | | | | | |
| Author, year | Objective | | Design | | Context | | Theory | | Sampling | | | Data collection | | Analysis | | Verification | | Conclusion | | Reflexivity | | Score |
| Tolstrup et al., 2023 | 2 | | 2 | | 2 | | 1 | | 2 | | | 2 | | 2 | | 0 | | 2 | | 2 | | 0,85 |

**Supplementary table 3. Studies using EORTC QLQ C30 or RAND/MOS Health Survey Short Form and reporting on subscales**

| **Studies using EORTC QLQ C30** | | | | | | | | |
| --- | --- | --- | --- | --- | --- | --- | --- | --- |
| **Author** | **Physical functioning** | **Emotional functioning** | **Social functioning** | **Role functioning** | | **Cognitive functioning** | **Symptom scales** | **Global Health Status/ Quality of life** |
| Esbensen et al. | Stable | Increased | Stable | Stable | | Stable | Nausea and vomiting: reduced  Fatigue, insomnia and pain: stable | Stable |
| Kirkhus et al. | Declined* | Not measured | Not measured | Not measured | | Not measured | Not reported | Declined* |
| Puts et al. | Stable | Increased | Stable | Stabel | | Stable | Stable | 22,6% declined, 41,9% stable, 35,5% improved |
| Kaufmann et al. | Decline | Stable | Stable | Decline | | Stable | worsening of insomnia** | Stable |
| Ronning et al. | Stable | Increase to 3m, decrease to 22m | Not measured | Not measured | | Not measured | Not measured | Increase to 3m, decrease to 22m |
| **Studies using RAND/MOS Health Survey Short Form** | | | | | | | | |
| **Author** | **Physical Component Subscale (PCS)** | | | | **Mental Component Subscale (MCS)** | | | |
| Taylor et al. | Decline*** | | | | Stable | | | |
| Jayadevappa et al. | Vitality (subscale of PCS) improved over time  Other subscales were stable | | | | Social function (subscale of MCS) improved over time  Other subscales were stable | | | |
| Reeve et al. | Decline | | | | Only the scores in MCS for lung, colorectal, and prostate cancer patients showed statistically significant decreases | | | |
| Posielski et al. | Stable from 0-12-24 months, decline from 24-36 months and stable rom 36-48-60 months. | | | | Stable from 0-12-24 months, decline from 24-36 months and stable rom 36-48-60 months. | | | |

*Significant, but not clinically relevant Blue: stable trajectory Abbreviations:

**Non-significant, but clinically relevant Red: decline in quality of life in comparison to baseline PCS: Physical Component Subscale

## ***Non-significant, no mention of clinical relevance Green: increase in quality of life in comparison to baseline MCS: Mental Component Subscale

**Supplementary table 4. Data extraction table**

| **Author; year** | **Quality of life Instrument** | **Number of measurements** | **Reported global quality-of-life scores and other quality-of-life domains if reported** | | | | | | | **Summary of results** |
| --- | --- | --- | --- | --- | --- | --- | --- | --- | --- | --- |
|  |  |  | **Baseline** | **During treatment period** | **Short term follow-up (FU)** | | | **Long term follow-up (FU)** | |  |
|  |  |  |  |  | **3 months** | **6 months** | |  |  |  |
| Baziliansky et al., 2023 | The Control, Autonomy, Self-Realization and Pleasure-12 Scale  Range: 12-48. Higher score = higher QoL | 4 | This study included Waves 1, 2, and 4 through 6 of the SHARE study  Waves 1 and 2 (collected in 2004–2007) were integrated to construct the baseline.  Wave 4 data were collected in 2008 and 2009 (designated as Time 1), and Wave 5 (Time 2) and Wave 6 data (Time 3) were collected in 2013 and 2015, respectively  Only participants with <20% missing data were included | | | | | | | Four quality-of-life trajectories emerged, but all trajectories were stable across the timepoints: % persons in each group.   - High (30,9%) - Medium-high (39,1%) - Medium-low (23%) - Low (7,1%) |
| Cummings et al., 2022 | The Quality of life  in Adult Cancer Survivors (QLACS)  Higher scores = poorer QoL, range 0-100, mean and median | 8 | Baseline = pre-surgery  QLACS: 67  88% retention | / | Not reported | | 9 months: Not reported | | 15 months  Not reported  24 months  Not reported  3y  Not reported  4y  Not reported  5y  Not reported  5y: 43% retention | Quality of life worsened over 9 months following surgery and then improved to better than pre-treatment level at 15 months and levelled off after. Best quality of life between 15 and 24 months. |
| De Boer et al., 2020 | EORTC QLQ C-30  EORTC QLQ-BR23  Higher score = better QoL  Mean values, range 0-100 | 3 | Baseline = diagnoses of metastases, disease progression or follow-up visit:  Global quality of life: 73,7 | / | Global quality of life: 72,1  90,6% retention | | Global quality of life: 72,9  84,9% retention | | / | No significant longitudinal changes were found. quality of life remained stable.  Only a not clinically relevant increase in loneliness between baseline and 6 months. |
| Decoster et al., 2018 | EORTC QLQ v-30: global health status/quality of life subscale  Higher score = better QoL  Mean values, range 0-100 | 2 | Baseline: at diagnosis or at disease progression/relapse, when a cancer treatment decision had to be made  Not reported | / | Not reported  76% retention | | / | | / | HRQoL improved with ≥10 points in 35% of the patients with a mean improvement of 29,75. HRQoL declined with ≥10 points in 28,2% with a mean decline of 29.4. |
| Esbensen et al., 2007 | EORTC QLQ C-30  Higher score = better QoL  Mean values, range 0-100 | 3 | Baseline: time of diagnosis  Global quality of life: 75  Emotional functioning: 75  Nausea & Vomiting: 0 | / | Global Quality of life: 66, 67  Emotional functioning: 83,33  Nausea & Vomiting: 0 | | Global quality of life: 66,67  Emotional functioning: 83,33  Nausea & Vomiting: 0  74% retention | | / | Global health status/quality of life showed no significant difference over 6 months. In 30,7% Quality of life deteriorated by > 10 units, in 69,3% no significant difference over the 6 months. Emotional functioning increased between baseline and 3 months. Nausea and vomiting reduced between baseline and 3 months and between baseline and 6 months. |
| Ikander et al., 2021 | FACT-G  Mean scores.  Range: 0-108, higher score = better quality of life | 3 | Baseline: before 1^st^ cycle of palliative chemotherapy  QOL: 73,2  Physical Wellbeing (WB); 20,5  Social WB: 19,9  Emotional WB: 15,5  Functional WB 16,5 | At 2^nd^ cycle of chemo (3 weeks after baseline)  Physical WB: 19,9  Social WB: 19,5  Emotional WB: 15,8  Functional WB: 17,8  92% retention rate  At 3^rd^ cycle of chemo (6 weeks after baseline)  QOL: 70,5  Physical WB: 19,1  Social WB: 19,7  Emotional WB: 14,7  Functional WB: 17,1  83% retention rate | / | | / | | / | Mean quality of life scores significantly decreased over the course of palliative chemotherapy. However, this decrease was not clinically meaningful.  Physical wellbeing, social wellbeing and functional wellbeing: no change  Emotional wellbeing: significant decrease |
| Jayadevappa et al., 2006 | University of California at Los Angeles’  Prostate Cancer Index (UCLA-PCI)  Medical Outcome Survey Short Form (MOS SF-36)  Higher score = higher quality of life. Range 0-100  Mean change scores: lower score is better functioning. | 4 | Baseline: at diagnosis  Caucasian:  PF: 62f4  RP: 76,8  RE: 85,9  V: 67,3  MH: 81,8  SF: 89,3  BP: 82,7  GH: 70  African American (AA):  PF: 52  RP: 61,2  RE: 70,2  V: 60,6  MH: 75,8  SF: 79,8  BP: 79,9  GH: 58,9 | / | Mean change baseline -> 3m  Caucasian:  PF: 4,5  RP: 19,7  RE: 5,9  V: 6,9  MH: 3,4  SF: 11,7  BP: 8,5  GH: 1,18  African American (AA):  PF: 4,8  RP: 12,6  RE: 1,8  V: 10,9  MH: -2,3  SF: 8,04  BP: 11,9  GH: 5,7  91% retention | | Mean change baseline -> 6m  Caucasian:  PF: 1,1  RP: 5,6  RE: 4,3  V: -0,04  MH: 0,59  SF: 6,5  BP: 3,7  GH: 1,99  AA:  PF: 6,1  RP: 7,6  RE: 5,8  V: 6,3  MH: -1,9  SF: 3,4  BP: 14,3  GH: 3,08  86% retention | | Mean change baseline -> 12m  Caucasian:  PF: 1,9  RP: 5,3  RE: -0,24  V: 1,04  MH: -0,91  SF: 3,1  BP: 1,3  GH: 2,9  AA:  PF: 1,5  RP: 12,4  RE: 14,1  V: 6,1  MH: -1,7  SF: 5,5  BP: 12,1  GH: 1,74  85% retention | Caucasians:  Role physical (RP) improved over time (baseline to 12 months). Role emotional (RE) improved over time. Vitality (V) declined at 3 months, improved 6 months, and somewhat declined at 12 months. Mental health (MH) score declined at 3 months, improved at 12 months. Social function (SF) lower than baseline at 3 months but improved at 6 months. Bodily pain improved over time. General health (GH) declined slightly over time.  African Americans: role physical improved at 6 months, declined at 12 months. Role emotional declined over time. Vitality improved over time. Mental health stayed higher over time than at baseline, although difference was smaller at 12 months. Social function lower than baseline at 3 months but improved at 6 months. Bodily pain was stable. General health improved over time. Bowel function improved over 12 months. Bowel bother improved greatly by 12 months.  Both groups:  Urinary function at 12months was lower than baseline (but more decline among AA). Sexual function was lower at 12 months. Urinary bother improved at 12 months. Sexual bother declined at months and improved at 12 months |
| Kaufmann et al., 2015 | EORTC QLQ C-30  EORTC QLQ-ELD14  Range 0-100, higer score = better outcomes.  Mean scores | 3 | Baseline = at the start of radiotherapy  Not reported | At the end of therapy: Not reported  78% retention | / | | Not reported  60% retention | | / | Between baseline and end of therapy: no statistically significant changes in any HRQoL domains. Although some small clinically relevant changes were seen in some domains (Role function and social function improved, cognitive function deteriorated)  From baseline to 6 months: (1) statistically significant deterioration in physical and role functioning, clinical relevance was small and moderate (respectively)  (2) worsening of insomnia was moderate clinically relevant, but not significant.  (3) deterioration in future worries, burden of illness and family support. Large clinical relevance for family support and moderate for other two.  (4) emotional and cognitive function deteriorated non-significant but clinically relevant |
| Kirkhus et al., 2019 | Physical function, global quality of life and symptom scales of EORTC QLQ C-30 | 4 | Baseline = at diagnosis  Not reported | / | 2 & 4 months: not reported  89,8% retention at 2m & 85,6% retention at 4m | | Not reported  75,8% retention | | / | Physical function and global quality of life declined over time significantly, but at none of the time points the change was clinically relevant (not more than 10 point) |
| Litwin et al., 2001 | RAND 36-item Health Survey (SF-36)  Range 0-100, higher=better outcome. Difference from 6 to 8 is considered clinicaly relevant. Mean scores. | 1-5 | Every 3 months until death. | Not reported  100% retention (participants needed only 1 completed questionnaire) | | | | | | Significant decrease in all 8 SF-36 domains in last 12 months of life.  Most substantial deteriorations were in role limitations due to physical problems (29 points), emotional problems (26 points), physical problems (24 points), social function (23 points) and bodily pain (18 points). Smallest decrease was in well-being (10 points). Also significant decrease in role limitations due to emotional problems and energy fatigue. |
| Maurer et al., 2021 | EORTC QLQ-C30  Range 0-100. Mean scores.  High scores quality of life/functional outcomes = better. High scores in symptom scales = worse | 5 | Baseline = before diagnosis  Not reported | Not reported | / | | / | | 1y:  Not reported  5y:  PF: 75,19  RF: 70,27  EF: 70,44  CF: 78,68  SF: 81,81  Appetite loss: 7,66  Financial difficulties: 12,74  Retention 61%  ≥10y:  PF: 71,59  RF: 69,98  EF: 72,76  CF: 79,41  SF: 80,73  Appetite loss: 10,7  Financial difficulties: 8,82  45% retention | Lowest quality of life during treatment. Highest 1 year before treatment, after treatment: increase after 1 year; than more stable quality of life but never as high as before diagnosis.  Role, emotional, cognitive, and social functioning: no significant longitudinal results. Significant, but trivial clinically relevant decline in physical functioning between first (5 years after) and second (10 years after) follow-up. Financial difficulties had a small decrease and appetite loss a small increase between FU1 (5y) and FU2 (10y), both significant but small clinical relevancy. |
| Melmed et al., 2002 | RAND 36-item Health Survey 1.0 (SF-36)  Range 0-100. Higher scores = better outcomes. Mean scores. | 13 | Baseline (before start of therapy), 3 months-interval for the first year and at 6-months interval for up to 5 years:  Not reported | / | Not reported | | Not reported | | Not reported  63,9% retention | HRQoL showed declining trend in the last year of life (non-significant). Significant declines were noted in   - Wellbeing in patients who are married - Physical function in patients who are single - General health perceptions, social function, and emotional well-being in those with at least a college degree - Role limitations due to emotional functioning, social functioning, and general health perceptions in those with annual household incomes over 30 000 dollars   Trends towards declines in both physical (PCS) and mental (MCS) summary scale but not significant.  Affluent patients worse declines in MCS (significant), while lower income patients trend toward lower PCS (non-significant).  Non-significant:   - PCS slower decline in married men, and better in men with higher incomes   MCS worse in men with higher incomes. |
| Mian et al., 2020 | FACT-G  Gynaecologic Oncology Group-  Higher scores = better overall Quality of life. Median scores | 2 | Baseline: within 3 months of diagnosis  PWB: 21  SWB: 24  EWB: 18  FWB: 15,7  Quality of life total: 75 | / | / | | PWB: 20  SWB: 24,5  EWB: 19,1  FWB: 20,5  Quality of life total: 85,5  90% retention | | / | No statistically significant changes in quality of life overall or subscales of FACT-G expect for emotional well-being which improved. Using the Minimall important difference cut-off of 5 for FACT-G, 69,4% of patients showed stable or improved quality of life. |
| Montroni et al., 2022 | EQ-5D-3L  Mean scores. Range: -0,111 (worse than death) to 1.000 (best health). VAS: 0-100 | 3 | Baseline: before surgery  EQ5D: 0,79  VAS: 69,9 | / | EQ5D: 0,79  VAS: 71,3  80,9% retention | | EQ5D: 0,82  VAS: 73,3  81,7% retention | | / | Most patients showed stable quality of life at 3 months compared to baseline and an improvement of quality of life at 6 months. Pain and depression score improved. But mobility, self-care, and ability to perform usual activities showed lower scores at 6 months. |
| Pivodic et al., 2021 | Physical, social, role, emotional functioning of EORTC QLQ C30  EORTC: higher scores = better outcomes  Mean scores. | 5 | Baseline: around diagnosis  Not reported | / | / | | Not reported | | 1y  Not reported  3y  Not reported  5y  Not reported  100% retention | Physical functioning and role functioning significantly declined towards death and depressive symptoms increased.  Also small and non-significant declines in emotional and social functioning. Higher initial score of social functioning is associated with a more rapid decrease. |
| Posielski et al., 2021 | RAND 36 Item Short Form Health Survey (SF-36)  SF-36: range 0-100, higer score = better outcomes. Subscale for mental (MCS) and physical (PCS) domain. | 10 | Baseline: pre-treatment  PCS: 52  MCS: 54 | / | Not reported | | Not reported | | 9m, 12m, 24m, 30m, 36m, 48m and 60m: Not reported  44,5% retention | *Summary results are written based on figure 2 in article*. Urinary function, sexual function, bowel function, hormone function and hormone bother remained stable over time. Urinary bother decreased linear from 24 to 60 months.  Sexual bother declined from 12 to 48 months but showed delayed improvement at 48 to 60 months. Bowel bother decreased from 48 to 60 months.  PCS & MCS score declined from 24 to 36 months and remained stable from 36 till 60 months. |
| Puts et al., 2011 | EORTC QLQ C-30  Range 0-100. Higher scores = better quality of life. Median scores | 6 | Baseline: before (any) treatment  Global quality of life: 66,7  EF: 80,6  PF: 93,3  RF: 100  CF: 100  SF: 100 | / | 1,5m  Global quality of life: 66,7  EF: 83,3  PF: 86,7  RF: 83,3  CF: 83,3  SF: 83,3  90,2% retention  3m  Global quality of life: 66,7  EF: 83,3  PF: 86,7  RF: 100  CF: 91,7  SF: 100  86,6% retention | | 4,5m:  Global quality of life: 66;7  EF: 83,3  PF: 86,7  RF: 100  CF: 100  SF: 100  83% retention  6m:  Global quality of life: 75  EF: 91,7  PF: 86,7  RF: 100  CF: 100  SF: 100  81,3% retention | | 12m:  Global quality of life: 75  EF: 91,7  PF: 86,7  RF: 100  CF: 100  SF: 100  69,6% retention | Little change in median scores over time. Between baseline and 12-monht FU: 23,1% declined in global health status/quality of life, 43,6% remained stable and 33,3% improved. Emotional functioning increased from baseline to 12 months. |
| Reeve et al., 2009 | SF-36  Range 0-100. Higher scores = better quality of life. Mean scores | 2 | Baseline: before diagnosis  PCS: 40,8-45,2  MCS: 51-54 | / | / | | / | | 2y (0-32m after diagnosis, mean of 12,4m): not reported  100% retention | Statistically greater decline in physical health summary scores from baseline to follow-up for cancer patients in comparison with healthy controls. + greater decline in all physical health domains except for bodily pain. The mental health summary score only decreased significantly for lung, colorectal and prostate cancer patients. Large decrease in social function ad lowered vitality. |
| Ronning et al., 2016 | EORTC QLQ C-30  Range 0-100. Higher scores = better Quality of life. Mean scores | 3 | Baseline: before surgery  Global quality of life: 66,1  PF: 80,9  EF: 81,1 | / | Global quality of life: 78,3  PF: 75,7  EF: 89,2  76,7% retention | | / | | 16-28m FU:  Global quality of life: 69,3  PF: 77,6  EF: 83,5  46,1% retention | Statistically significant increase in emotional functioning and quality of life from baseline to 3 months, but again a decrease to follow-up.  No significant increase in scores from baseline to 3 months for physical functioning. |
| Scheepers et al., 2023 | EORTC QLQ C-30  Range 0-100. Higher scores = better Quality of life. Means scores | 3 | Baseline:  Before surgery  Summary score quality of life: 87,3  Global quality of life: 76,6  PF: 85,5  RF: 87  EF: 82,7  CF: 91,7  SF: 90,8 | / | Not reported  95,2% retention (in total cohort, not 70+) | | / | | 12m:  Not reported  70,7% retention (in total cohort, not 70+) | 87% of the patients recovered to baseline scores of quality of life after 12 months, with decline at 3 months. Other 13% were non-resilient (clinically relevant decline at 3 months that continued until 12 months) or had a late decline (= clinically relevant decline at 12 months without decline at 3 months). In patients with adjuvant therapy: 32% of the patients were resilient (meaning the decline at 3 months was clinically relevant) and 16% had a late decline. |
| Taylor et al., 2023 | MOS SF-12  Range 0-100, higer score = better outcomes | 6 | Baseline: study entry (already got diagnosis, treatment status did not matter)  Not reported | / | / | | Not reported | | 18, 36, 54, 72m:  Not reported  89% retention at 24m | PCS12 scores tended to decline over time, but this was not statistically significant. MCS12 tended to be stable over time. |
| Tolstrup et al., 2023 | EORTC QLQ c-30  Interview  EORTC: Range 0-100, higer score = better outcomes.  Mean scores | 2 quantitative  2 qualitative | Baseline: before start or within first week of treatment  Global quality of life: 28,56 | / | Global quality of life 54,68  81% retention | | / | | / | 62% showed a minimally important improvement in quality of life at follow-up, 10% showed no change and 10% showed clinically important declined in quality of life.  Overall: significant and clinically relevant improvement of quality of life. Except for people receiving palliative treatment.  Results from interviews:  4 themes:  Adjusting mentally to cancer: mental health improved in comparison to baseline. Were more at ease, less anxiety, and less worry about the future.  Changing physical health: more tired than before the cancer diagnosis. Despite, they were satisfied with current circumstance. Positive change since baseline.  Influence of comorbidity: influence of comorbidity at baseline remained large at follow-up.  Living with side effects: tired, infections, nausea alopecia, constipation, weight loss, dysphagia, pain, and skin toxicity. ¼ did not have side effects, made dealing with treatment easier. Gradually regaining HRQoL from before diagnosis  Combined results from interview and questionnaires:  Poor HRQoL at treatment initiation, HRQoL heavily impaired by diagnosis. Substantial improvement at follow-up |

*Abbreviations:*

*QoL: quality of life HRQoL: health related quality of life WB: wellbeing*

*FU: follow-up PCS: physical component subscale*

*PF: physical functioning RP: role physical RE: role emotional*

*V: vitality MH: mental health SF: social functioning*

*BP: bodily pain GH: general health CF: cognitive functioning*

*PWB: physical wellbeing SWB: social wellbeing EWB: emotional wellbeing*

*FWB: functional wellbeing*
